# Zinc Supplementation Improves Texture, Oxidative Stability of Caciotta Cheese and Reduces Biogenic Amines Production

**DOI:** 10.3390/ani14111642

**Published:** 2024-05-31

**Authors:** Carmela Sorice, Andrea Ianni, Francesca Bennato, Mirella Bellocci, Valentina Pavone, Lisa Grotta, Clemencia Chaves López, Giuseppe Martino

**Affiliations:** 1 Department of BioScience and Technology for Food, Agriculture and Environment, University of Teramo, Via Renato Balzarini 1, 64100 Teramo, Italy; csorice@unite.it (C.S.); fbennato@unite.it (F.B.); valentina.pavone@studenti.unite.it (V.P.); lgrotta@unite.it (L.G.); cchaveslopez@unite.it (C.C.L.); gmartino@unite.it (G.M.); 2 Istituto Zooprofilattico Sperimentale dell’Abruzzo e Molise “G. Caporale”, Campo Boario, 64100 Teramo, Italy; m.bellocci@izs.it

**Keywords:** zinc oxide, milk, caciotta cheese, biogenic amines, oxidative stability, texture, quantitative descriptive analysis, fatty acid profile, lactating cow, NSLAB

## Abstract

**Simple Summary:**

Zinc is an essential nutrient for animals, the effects of zinc depend on factors such as level of supplementation and animal health. However, few studies have investigated its impact on cheese quality. This study aimed to evaluate the effect of Zinc Oxide (ZnO) supplementation in the diet of lactating Friesian cows on chemical composition, zinc content, fatty acid and proteic profile, ammine content, pH, a_w_, texture, and sensory profile of cheese. Zinc is known for its antimicrobial properties and can reduce microbial growth during ripening period. It also has antioxidant abilities and could reduce lipid oxidation by affecting oxidative stability. The results showed that ZnO supplementation improved the texture, the sensory profile and stability of caciotta cheese and reduced the production of biogenic amines. ZnO supplementation could be a promising strategy for improving the quality and shelf-life of caciotta cheese.

**Abstract:**

Zinc is essential for animals, playing a vital role in enzyme systems and various biochemical reactions. It is crucial to ensure a sufficient intake of zinc through the diet to maintain efficient homeostasis. Only few studies on zinc effect in cow lactating diet evaluated the effects on milk and cheese quality, with conflicting findings. 24 cows of the Friesian breed were divided into two groups (CTR: control and TRT: treated group). Cows were selected for age, body weight, parity and phase of lactations (mid lactation, 140–160 days). CTR diet contained 38 mg/kg of Zn and TRT diet was supplied with 120 mg/kg of complete feed for 60 days. The objective of current investigation was to evaluate the impact of a dietary Zinc Oxide (ZnO) integration of lactating Friesian cows on chemical composition, zinc content, fatty acid and proteic profile, ammine content, pH, a_w_, texture, and sensory profile of cheese and to improve the chemical-nutritional quality of milk and cheese. The results showed that ZnO supplementation reduced mesophilic aerobic bacteria and Presumptive *Pseudomonas* spp. growth, proteolysis, biogenic amines content, lipid oxidation, odour intensity and sour and increased hardness, gumminess, chewiness, elasticity of cheese. Biogenic amines are considered an important aspect of food safety. ZnO integration in cow diet could represent a promising strategy for improving the quality, the safety and shelf-life of caciotta cheese.

## 1. Introduction

The nutritional quality of milk and cheese depends on several factors (animal breed, ruminal fermentation, health status, stage of lactation, feeding regimen, and seasons). Microelements are fundamental for animal nutrition. Zinc is widely utilized in biological systems, making it one of the most employed trace elements. Over 2700 enzymes, such as hydrolases, transferases, ligases, isomerases, and lyases, contain zinc in their structure. Remarkably, Zn is present in nearly every cell of the body. Apart from contributing to the stability and integrity of biological membranes and ion channels, zinc serves as an intracellular regulator and imparts structural support to proteins during molecular interactions. Furthermore, it plays a crucial role as a structural component in nucleic acids and other gene-regulating proteins [1].

Zn is essential for animals, playing a vital role in enzyme systems and various biochemical reactions like protein synthesis and carbohydrate metabolism. A deficiency in Zn can cause reduced growth, skin issues, lethargy, weakness, and increased susceptibility to infections. An adequate Zn status relies on proper dietary supply and efficient homeostasis, as zinc is an essential trace element necessary for enzymatic, structural, and regulatory functions [2]. Given the scarcity of body reserves, it is crucial to ensure a sufficient intake of Zn through the diet to maintain efficient homeostasis for its optimal utilization and distribution.

The digestibility and fermentation of nutrients can be affected by the solubility of specific trace minerals in the rumen. Zn is a microelement that can influence ruminal fermentation and digestion in ruminants. The degree of binding of Zn to ruminal solid digesta can be influenced by the origin of trace minerals [3].

Inorganic zinc, specifically zinc oxide and zinc sulphate, is widely used as a Zn supplement for animals. Petrič et al., (2021) [4] reported that the impact of zinc organic supplementation on ruminal fermentation is dose dependent. Low doses of Zn (20–70 mg Zn/kg diet) have a weak effect on ruminal fermentation, while higher doses (250–1142 mg Zn/kg diet) can significantly affect ruminal protozoal populations and protein degradation. Interestingly, the ruminal microbiota may have a specific requirement for Zn supplementation that does not cause any adverse effects on digestion or animal health, nor does it result in a major shift in the ruminal bacterial community.

Limited studies assessed Zn effect on cheese quality, with conflicting findings. Some studies report positive effects on milk composition and zinc content after zinc supplementation [5], while other studies found no significant effects [6,7,8].

Animal food products typically contain more Zn than vegetables. Dairy products can significantly contribute to meeting the recommended zinc intake. Cow cheeses generally have the highest Zn content, ranging from 1.83 to 7.75 mg/100 g of cheese. Sheep cheeses follow with 1.34 to 3.69 mg/100 g, while cheeses made from mixed milk have a Zn content ranging from to 0.39 to 4.54 mg/100 g [9]. 

The aim of the present work was to evaluate the impact of a dietary Zn integration of lactating cows on chemical composition, Zn content, fatty acid, proteic and microbial profile of milk and cheese and ammine content, pH, a_w_, texture, sensory profile, and oxidative stability of caciotta cheese and to improve the chemical-nutritional quality of milk and cheese, as well as their safety and stability.

## 2. Materials and Methods

### 2.1. Experimental Design

A process of selection was carried out to randomly allocate 24 cows of the Friesian breed into two groups. The aim was to ensure uniformity in terms of age, body weight, number of lactations, and lactation phase (mid lactation, 140–160 days). The experimental group (TRT) and control group (CTR) comprised both of 12 cows. CTR was provided with a basal diet without any additional supplements. On the other hand, TRT group was given a diet supplemented with 82 mg of zinc for kg of complete feed (totally 120 mg/kg) from zinc oxide (ZnO) powder. This supplementation lasted for 60 days.

During this time, the animals received a combination of unifeed, hay and concentrate. For the administration of zinc, a different formulation of the concentrate was provided. To the CTR group was supplied by the farmer’s standard concentrate while for the TRT group the concentrate was formulated by adding ZnO powder for a total of 120 mg/kg of Zn. Both groups continued to follow the same diet with the only difference in the content of Zn. The concentrate ration was administered manually to the animals, with each cow receiving 500 g, which was subtracted from the total TMR ration formulation.

It is important to note that the standard diet adequately fulfilled the nutritional requirements of lactating cows, while the TRT group received an additional supplementation of 80 mg of Zn. The dosage given adhered to the instructions specified in Regulation (EC) No. 1831/2003 of the European Parliament and the Council dated 22 September 2003 concerning additives utilized in animal nutrition (European Commission, 2003) [10]. The animals were fed a daily ration of approximately 22 kg of dry matter per head in the form of a total mixed ration (TMR) including 500 g of concentrate manually administered. TMR composition was determined based on the guidelines outlined in the eighth edition of Nutrient Requirements of Dairy Cattle (2021) [11] as indicated in Table 1 and the detailed composition of concentrate is given in Table 2.

In this study, the monitoring of zinc supplementation was conducted to ensure that it remained within the prescribed limit of 120 mg/kg of complete feed, as set by the European Union (European Commission; EC 2016/1095) [12].

### 2.2. Samples Collection (Feed, Milk, Cheese) and Cheesemaking Production

Total mixed ration and the two different concentrates administered were collected at 0 time and 60 days and analyzed to determine the chemical composition and the zinc content. At 60 days, fresh milk (100 L), collected in two different milking, was immediately used for cheese production and to determine the chemical composition. Some milk aliquots were frozen at −20 °C for further analysis. The bulk milk of each group was used for caciotta cheese production. The process was carried out in a local dairy. The milk was subjected to heat treatment at 55 °C for 5 min. It was then quickly cooled to 40 °C and transferred to a boiler, where rennet (30 mL/100 L) was added. The rennet used was a mixture of 80% chymosin and 20% pepsin, with a strength ratio of 1:10,000 (microMilk srl, Cremosano (CR) Italy). No starter cultures were added. Milk coagulation was achieved in 40 min at 38–40 °C. The curd was cut twice into pieces of medium grain. Between the first and the second cut, the curd was left to stand 5′. The extraction took place after the second cut, and the cheese was pressed and formed into round molds with an average weight of 1.3 ± 0.1 kg. The whey was drained and the cheese dried at 37 °C for 3 h. After removing the cheese from the molds, the salting phase was done with a 20% solution of NaCL water. The maturation process was conducted in a room with controlled temperature (10 ± 0.5 °C) and 85% relative humidity. The cheese-making process produced 12 forms of 1.3 kg ± 0.1 (6 from the control milk and 6 from the treated milk). After 1 and 30 days of maturation, the cheese was analysed for T0 and T30, respectively.

### 2.3. Total Mixed Rations, Concentrate, Milk and Cheese Chemical Composition

Dry matter, crude protein, ether extract, crude fiber and ash of TMR and concentrate were determined according to AOAC methods [13] International by method 930.15, 954.01, 920.39, 962.09 and 942.05, respectively. The chemical composition of milk, including fat, proteins, caseins, lactose, and urea content, was analyzed using MilkoScan FT 6000 (Foss Integrator IMT; Foss A/S, Hillerod, Denmark). The pH values of cheese were determined by a portable pH meter (Crison, Barcelona, Spain). Water activity (a_w_) was determined at 25 °C by Aqualab 4TE (Meter Group, Pullman, WA, USA). The moisture content was calculated using the AOAC methods [14] while the lipid fraction was extracted according to the official AOAC method [13]. The method involves extraction by acid hydrolysis. Three grams of cheese after adding 20 mL of HCl (25%) were homogenized. The samples were placed in a thermostatic bath and subjected to a temperature of 100 °C. After the acid deproteinization phase, the lipid extraction was carried out with a solution of 80 mL of ethyl ether and petroleum ether (1:1). The supernatant was recovered in a round-bottom flask to dry out the solution in Strike-Rotating Evaporator at 60 °C (Steroglass s.r.l, Perugia, Italy). The flasks were placed in a dessiccator for one hour. The samples were weighed after cooling at room temperature. Total lipid content values were reported on a dry matter basis.

### 2.4. Zinc Content of Total Mixed Rations, Concentrate, Milk and Cheese

The stock solutions of zinc (Zn) and gallium (Ga) at 1000 mg/L in 5% (*v*/*v*) HNO_3_, tellurium (Te) at 1000 mg/L in 20% (*v*/*v*) HNO_3_ and nitric acid >60%, ultrapure for trace analysis were provided by Merck KGaA, Darmstadt, Germany. All solutions were prepared using Type I water (resistivity 18.2 MΩ·cm, ELGA LabWater PURELAB Option-Q water purification system, High Wycombe, UK). The samples under analysis were homogenized and weighed in a PTFE/TFM (polytetrafluoroethylene, modified) container, treated with 4 mL of nitric acid, and then mineralized using an ultraWAVE SRC microwave digestion system with a single reaction chamber (Milestone Srl, Sorisole (BG), Italy) according to the standardized method: UNI EN 13805:2014. The amount of processed sample varied depending on the matrix, being 2.0 ± 0.2 g for milk, 1.0 ± 0.2 g for cheese, and 0.5 ± 0.1 g for feed and unifeed, the latter previously dried in an oven. The resulting solutions were diluted to 15 mL with water and analyzed using a Q-ICP-MS Nexion 2000 (PerkinElmer, Waltham, MA, USA), in accordance with the FDA U.S. FOOD & DRUG ADMINISTRATION method 4.7—Version 1.2 (February 2020) for milk and cheese, and the UNI EN 17053:2018 method for animal feeding stuffs. For the determination of Zn, a metal subject to isobaric interferences, a collision cell (KED) was used, which exploits the ability of helium gas to remove typical interferents of medium-low masses through collision. The acquired isotopic masses were ^67^Zn, ^71^Ga, and ^130^Te. Quantitative analysis was obtained using a mixture of internal standards and a matrix calibration, which reproduces the same conditions as the real samples in the plasma, allowing the minimization of the matrix effect. The calibration curve was built using the matrix matching technique, where increasing concentrations of zinc were added to a series of aliquots of a solution matrix with a composition similar to that under analysis, but with a low content of the target analytes, in the presence of a fixed concentration of internal standards (Ga, Te). The obtained results were expressed in units of mg/kg for food and in mg/kg with a moisture content of 12%, for feeding stuff.

### 2.5. Fatty Acids Profile of Concentrate, Milk and Cheese

The extraction of fatty acids (FA) of concentrate was carried out by utilizing hexane. Conversely, the milk lipid fraction was examined in accordance with the AOAC official method [13]. The lipid fraction of the cheese was investigated according to the method described by [15], using a mixture of chloroform and methanol (2:1, *v*/*v*). The lipid extracts of concentrate, milk and cheese were subjected to transmethylation and the resulting FAME were separated following the procedure outlined by [16]. The detection of FAMEs was performed by a gas chromatography (GC) coupled with a flame ionization detector (FID) equipped with a capillary column (Restek rt-2560 Column, fused silica 100 m *×* 0.25 mm highly polar phase; Restek Corporation, Bellefonte, PA, USA). The identification of the FAMEs was achieved by the comparison of the retention times with some FAME analytical standards for concentrate (FAME Mix C8-C24 Supelco, Bellefonte, PA, USA) and milk (FIM-FAME-7-Mix; Matreya LLC, Pleasant Gap, PA, USA). The ChromeCard software (Version 2.12, 2015, Thermo Fisher Scientific, Waltham, MA, USA) was utilized to quantify peak areas, and the percentage of total FA was used to express the relative value of each individual FA. Monounsaturated fatty acids (MUFA), polyunsaturated fatty acids (PUFA), and saturated fatty acids (SFA) were calculated based on sum of the relative percentage values of each FA. To assess the Atherogenic and Thrombogenic Index (AI and TI), the formulas proposed by [17] were used to calculate the respective indices in milk and cheese. Additionally, the desaturation indices (C4, C16, C18) were calculated following the method proposed by [18].

### 2.6. Oxidative Stability of Cheese (TBARS)

The evaluation of oxidative stability was conducted to assess the ability of the spectrophotometric thiobarbituric acid reagent (TBARS) to determine the presence of malondialdehyde (MDA) in cheese samples at both 0 and 30 days. MDA, a highly reactive aldehyde formed through lipid oxidation, is frequently employed as an indicator of oxidative damage in cheese.

Fat oxidation of cheese was measured by extracting MDA from cheese according to the method of [19], with minor modifications. To prevent oxidation, after removing each sample from the freezer, 0.1% of butylated hydroxytoluene in methanol solution was mixed with 4.5 g of frozen cheese. The mixture was then homogenised with a high-speed homogenizer Ultra Turrax from IKA Werke (T-25, Staufen, Germany) in 45 mL of an aqueous solution of 7% trichloroacetic acid and subjected to distillation. 2 mL of each distillate were combined with an equal volume of a 0.02 M solution of thiobarbituric acid (TBA) in 90% acetic acid. The mixture was incubated in a thermostatic bath at 80 °C for 1 h. After 1 h of cooling, the absorbance of the reaction product (TBA-MDA) was measured using a spectrophotometer (Jenway, Essex, UK) at a wavelength of 534 nm. The concentration of oxidized lipids was determined by calculating micrograms of malondialdehyde per gram of cheese using a calibration curve.

### 2.7. Proteic Profile (SDS-PAGE) of Cheese and Milk

The milk samples were cooled, and skimmed after a centrifuge step at 4000 rpm for 15 min. The supernatant, after removal of the lipid part, was filtered and used to quantify the protein content by the Bradford method [20].

To extract protein, the procedure of [21] was utilized as follows. 1 g of cheese sample was dissolved in 20 milliliters of Tris-glycine 0.01 M, pH 8.3, and urea 6 M. The mixture was homogenized for 2 min. Subsequently, the extract was left to incubate for 2 h at 37 °C to aid in casein solubilization. Afterward, the solution underwent centrifugation at 10,000× *g* (4 °C) for 15 min. The resulting supernatant was then collected and passed through Whatman filter paper to eliminate fat and other insoluble solids. Bovine serum albumin (BSA) was used for calibration. The milk samples before being loaded onto a 12% polyacrylamide gel were diluted in a sample buffer (0.5 M Tris-HCl, pH 6.8; 2% (*w*/*v*) SDS; 7% (*v*/*v*) glycerol; 4.3% (*v*/*v*) β-mercaptoethanol; 0.0025% (*w*/*v*) bromophenol blue) and boiled for 5 min to denature the proteins. After the electrophoretic run, the gels were stained with an aqueous dye solution containing 40% (*v*/*v*) methanol, 10% (*v*/*v*) acetic acid and 0.1% Comassie Brillant Blue G-250 and bleached twice with the same solution without adding Comassie Brillant Blue G-250. Densitometric analysis was carried out by quantifying the % relative band of caseins and whey protein by using Image Lab^TM^ (Version 6.0.1 build 34, 2017, Bio-Rad Laboratories, Inc., Hercules, CA, USA) software.

### 2.8. Texture Analysis of Cheese

The textural properties of the samples were assessed under room temperature conditions (20 ± 1 °C) using an Instron Universal Testing Machine (Model 4452, Instron Ltd., Wycombe, UK) equipped with a 500 N load cell. A Texture Profile Analysis (TPA) test was conducted with a crosshead speed of 0.42 mm/s. Each sample, measuring 15 mm × 10 mm × 10 mm, was compressed by 30% of its initial height using a plunger with a circular surface (58 mm diameter). The compression was performed using a double-compression method with a 5-s delay between the first and second compression. Hardness, cohesiveness, and gumminess were determined following the methodology described by [22]. Chewiness (mJ) was calculated as Gumminess (N) × Elasticity (mm) and Elasticity (%) as (Distance 2)/(Distance 1) × 100.

### 2.9. Biogenic Amines (BAs) Content of Cheese

Five grams of cheese samples were subjected to determination of BAs (Cadaverine, Putrescine, Histamine, Tyramine, Tryptamine, 2-Phenylethylamine, Spermine, Spermidin). BAs extraction was performed following the procedure reported from [23]. A linear gradient (0.1 M ammonium acetate (A) and acetonitrile (B)) was utilized for chromatographic separation with a flow rate of 1 mL/min and a column temperature of 38 °C ± 0.1. Initially the mobile phase was constituted of 50% of A. This percentage linearly decreased up to 10% in 20 min. Subsequently, in 5 min this percentage increased up to 50% and was kept for 10 min. The separation was obtained by using a Supelcosil LC-18 column (25 cm × 4.6 mm, 5 µm). The identification of BAs was obtained by injecting each standard amines and their mixture at determined concentration (μg/mL) and comparing the standard amines solution retention time. To quantify BAs was prepared a calibration curve ranging to 10 from 100 μg/mL. An internal standard (I.S.) was added in each sample and the area of detected amines was relationated with the area of the I.S and the BAs concentration was extrapolated from calibration curve of each standard amines. The area of standard amines was also divided by I.S. area.

### 2.10. Microbiological Analysis of Milk and Cheese

Ten g of cheese samples were mixed with 90 mL of 0.85% (*w*/*v*) sterile physiological saline solution, and homogenized in a Stomacher Lab-blender 400 Circulator (Seward, Worthing, UK) for 2 min. To prepare serial dilutions was utilized 1 mL of previous solution for cheese while for milk, 1 mL was mixed with 9 mL of diluent. Mesophilic aerobic bacteria (MBA) were enumerated on PCA and incubated aerobically at 30 °C for 48 h instead mesophilic lactobacilli on Man Rogosa and Sharpe (MRS) and were cultivated in anaerobic conditions using anaerobic jars and BBL GasPak anaerobic system envelopes (Becton Dickinson, Franklin Lakes, NJ, USA) at a temperature of 30 °C for a duration of 48 h. *Pseudomonas* spp. were enumerated on Pseudomonas Agar Base Selective (PSA) according to ISO/TS 11059:2009 (IDF/RM 225:2009) aerobically at 30 °C for 48 h. PSA was supplemented with PP Supplement contains Primaricin (Natamycin) and Penicillin G. Enterococci were enumerated anaerobically on Slanetz Bartley Agar (SBA) according to ISO 7899-2 at 30 °C for 48 h. Enterobacteriaceae were enumerated by medium Violet Red Bile Glucose Agar (VRBGA), according to USP/EP/JP and ISO 21528 at 37 °C for 48 h. Coliform bacteria were isolated and enumerated anaerobically on Violet Red Bile Lactose Agar Selective (VRBLA) medium according to APHA and ISO 4832 at 37 °C for 48 h and *Staphylococcus aureus* on Mannitol Salt agar (MSA) at 30 °C for 48. MSA is a medium recommended by the American Public Health Association (APHA) for the identification, differentiation and enumeration of staphylococci based on their ability to ferment mannitol. *Salmonella* and *Listeria monocytogenes* isolation were carried out by ISO 6579 (ISO, 2002) and ISO 11290-1 (ISO, 2004), respectively. All media were purchased from © Liofilchem^®^. YPD was purchased from Sigma-Aldrich and supplemented with chloramphenicol. It was incubated at 25 °C for 72 h to enumerate mold and yeasts.

### 2.11. Quantitative Descriptive Analysis (QDA) of Cheese at 30 Days

A quantitative descriptive analysis (QDA) on control and experimental cheese was performed by using 23 attributes to describe the sensory profile of investigated cheese after 30 days of maturation: colour (yellow intensity), odour (intensity, mouldy, fruity, hay, grassy, butyric, acetic and propionic acid, rind), taste (acid, sweet, salty, bitter, spicy), texture (elasticity, hardness, gumminess, chewiness, graininess, solubility, moisture).

Flavour and texture attributes were selected as suggested by [24] with some modifications. The selection and evaluation of the panel adhered to the established global criteria (ISO 8586-1: 1993, ISO 8589:1998, ISO/DIS 13299:1998). These panellists demonstrated exceptional discernment, sensitivity, and uniformity in their assessments. The laboratory utilized for the sensory analysis adhered to the specifications outlined in ISO 8589 (1985). It was equipped with individual cabins and illuminated with white lighting (D65). The sensory evaluation was conducted in two separate sessions, one in the morning and another in the afternoon, following the guidelines set by ISO 6658 (1988). All samples were evaluated during both sessions. Before testing, the two samples were allowed to reach room temperature (20 °C) and each wheel of cheese was divided into two portions. One portion was left intact for visual evaluation, while the other portion was cut into cubes for assessing its colour, odour, taste and texture. The panellists evaluated the characteristics of control and treated cheese using a structured scale consisting of five points (ranging from 1 = extremely low to 5 = extremely high). They recorded their ratings on a paper scorecard and had the freedom to taste the cheese cubes as many times as they wished. To cleanse their palate between samples, mineral water and unsalted crackers were utilized.

### 2.12. Statistical Analysis

Analysis of Variance (ANOVA) was employed to examine the influence of zinc integration (Z), time (T) and their interaction (Z × T) in presence of four groups. Instead Student’s *t*-test was employed to assesses the difference between the means of two groups. The results were presented as means ± standard deviation for analyzed data by *t*-test and ±root mean square error (RMSE) for the results obtained from ANOVA. The HSD Tukey’s test was employed to examine the differences among group means and only the differences with a *p* < 0.05 or <0.001 were considered significant. The letters to differentiate groups are a result of interaction of treatment (Z) and time (T). The JMP Pro 14 program (SAS Institute, Cary, NC, USA) was utilized for statistical data analysis. Pearson’s Correlation was also carried out by XLSTAT to determine the correlation between some variables of cheese at 30 days.

## 3. Results

### 3.1. Total Mixed Ration Composition

Composition of total mixed ration administered to CTR and TRT groups (Table 1) did not show differences for % of ingredients.

### 3.2. Concentrate Chemical Composition and Fatty Acids Profile

Chemical composition of concentrate reported in Table 2. shows differences between concentrate of CTR and TRT group only for zinc content (mg/kg) (CTR: 38 vs. TRT: 120). No differences are present for dry matter (%), crude protein (%), crude fat (%), crude fiber (%), crude ash (%) and fatty acids (%).

### 3.3. Milk Chemical Composition

Zinc supplementation did not induce statistical differences for % of caseins, lactose, lipids, proteins and concentration of urea in milk chemical composition (CTR M and TRT M) obtained from CTR and TRT groups (*p*-value > 0.05). The same effect was reported for zinc content, pH and fatty acid profile (*p*-value > 0.05) (Table 3).

### 3.4. Cheese Fatty Acids Profile

In Table 4 are reported the percentage of fatty acids of cheese CTR and TRT at 0 and 30 days. No differences were identified in fatty acids profile of both cheeses during the ripening for the effect of treatment (Z), time (T) and their interaction (Z × T) (*p*-value: >0.05).

### 3.5. Dry Matter, pH, a_w,_ and Total Lipid Content of Cheese

CTR and TRT cheeses differed for dry matter (*p*-value < 0.001) and pH values (*p*-value < 0.05) at 0 day for the effect of treatment (Z). The differences were evident also at 30 days of ripening demonstrating the significant effect of time (T) (*p*-value < 0.001) for both parameters and also for total lipid content (*p*-value < 0.05). The treatment (Z) did not induce differences in a_w_, zinc and total lipids content for fresh cheese (CTR T0 and TRT T0). The effect of interaction of treatment and time (Z × T) was highlighted only for decrease of pH in CTR cheese from 4.85 to 4.24 (*p*-value < 0.001) while in TRT samples pH values did not change over the ripening period (Table 5).

### 3.6. TBARS Content of Cheese

**Figure 1:**
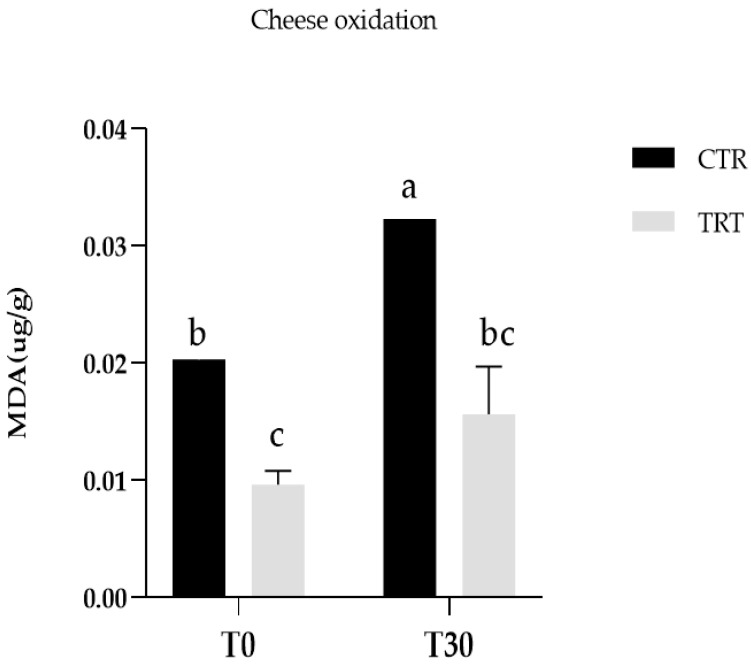
Lipid oxidation of control (CTR) and treated cheese (TRT) in two different stage of maturation (1 days: T0 and 30 days: T30). Different letters indicate statistically significant differences (*p*-value < 0.05). Error bars represent standard deviation.

Thiobarbituric acid reactive substances (TBARS) assay is a method to detect lipid oxidation. It measures malondialdehyde (MDA), which is a product of an endoperoxide of unsaturated fatty acids resulting from oxidation of lipid substrate. We detected significant differences between CTR and TRT cheese at 0 days. Meanwhile, TRT T0 did not show significant differences throughout the ripening period comparing to TRT T30. CTR T30 was significantly different from CTR T0 and TRT T30 (Figure 1).

### 3.7. Milk and Cheese Proteic Profile (SDS-PAGE)

**Figure 2:**
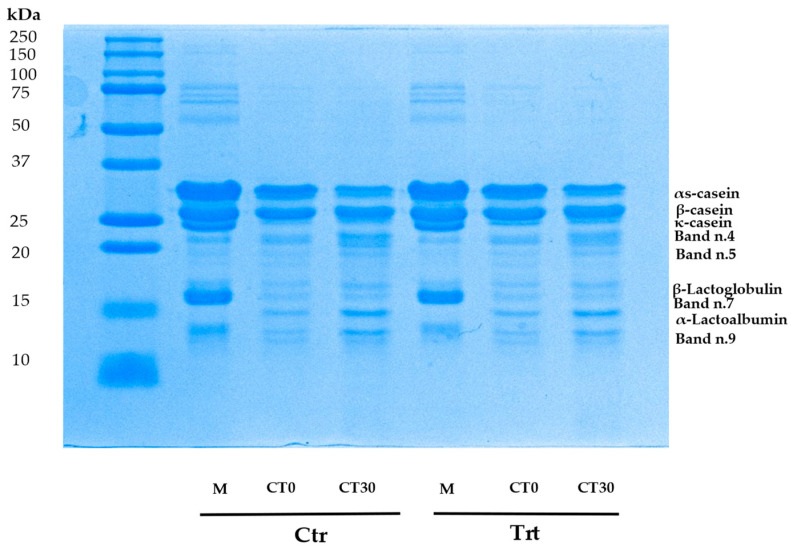
Representative image of SDS-PAGE analysis performed on milk before cheesemaking and on cheese at 1 (T0) and 30 (T30) days of ripening, obtained from milk of control and treated group (n = 3). Ctr: control; Ttr: treated; M = milk; CT0 = cheese at 1 day, CT30 = cheese at 30 days.

As reported in Table 6. analysis of milk proteins (CTR M and TRT M), expressed as relative % of band of α-casein, β-casein, k-casein, β-lactoglobulin, α-lactalbumin and other proteolitic fragments (band n.4, band n.5, band n.7) did not show differences (*p*-value > 0.05). An image of SDS-PAGE of milk and cheese proteic profile is visible in Figure 2.

Table 7 shows the relative % of bands corresponding to cheese identified proteins of CTR and TRT (α-casein, β-casein, k-casein, β-lactoglobulin and α-lactalbumin and other proteolitic fragments (band n.4, n.5 and n.7, n.9). In respect to milk proteins, we reported the band n.9 in cheese samples, which was not evident and measurable in milk. This band could be attributed to a proteolitic fragment. Effect of treatment (Z) (*p*-value < 0.05) was identified in relative % of α-caseins. This protein decreases in CTR and TRT cheese T30, but the reduction was higher in CTR T30. At 1 day no differences were detected (*p*-value > 0.05). The decrease of α-caseins is significant also for the effect of time (T) (*p*-value < 0.05) and for the interaction of treatment and time (Z × T) (*p*-value < 0.05), the same is reported for band n.7 (*p*-value < 0.05). This band, together with band n.4, increase at 30 days because of natural proteolysis in CTR and TRT cheese (effect of time (T), *p*-value < 0.001). Caseins are more susceptible to proteolitic events. In TRT cheese (T30), β- casein showed more resistance to these biochemical reactions, without significant changes. The opposite is detectable in TRT sample at the end of ripening period (T30) (*p*-value < 0.05) for the effect of time (T). The band corresponding to alpha lactalbumin (kDa: 12), a whey protein, shows a % increase at 30 days in CTR and TRT sample (*p*-value < 0.001). The increase is significant for the sample CTR at 30 days which changes from CTR at time 0. In TRT cheese the difference is less marked.

### 3.8. Texture Analysis of Cheese

Analyzing the texture of TRT were observed higher values of Hardness (*p*-value: < 0.001), Elasticity (*p*-value: < 0.05), Gumminess (*p*-value: < 0.001), and Chewiness (*p*-value: < 0.001) at 30 days due to the treatment effect (Z) in respect to CTR T0 and CTR T30. Hardness increased in TRT T30 (TRT T0: 2.91, TRT T30: 3.49) while in CRT T30 (CTRT T0: 2.45 CRT T30: 2.32) decreased as treatment effect (Z) (*p*-value: < 0.001) and interaction of treatment and time (Z × T) (*p*-value: < 0.05). The same trend of Hardness was observed for Chewiness (mJ) (CTR T0:154.02, CTR T30:135.51, TRT T0: 188.55, TRT T30: 211.02) (*p*-value: < 0.001) and Gumminess (N) (CTR T0:180.51, CTR T30: 166.22, TRT T0:215.47, TRT T30: 241.97) (*p*-value: < 0.001) due to zinc effect (Z) and zinc and time interaction (Z × T). Treatment (Z) induced an increase in Elasticity (%) at 30 days in CTR and TRT cheese (*p*-value < 0.05). Significant effect was observed also in a decrease of Cohesivity in TRT T30 (*p*-value < 0.05 for zinc effect (Z) and *p*-value < 0.001 for effect of time (T) and for their interaction (Z × T) (Table 8).

### 3.9. Biogenic Amines Content of Cheese

A chromatographic analysis was carried out to determine biogenic amines (BAs) in cheese CTR and TRT (T0 and T30). No BAs were detected in CTR T0 and TRT T0, while we quantified 3 BAs (putrescine, cadaverine and tyramine in CTR T30) (*p*-value < 0.05). BAs in TRT T30 were not detectable (LOD:10 μg/g) (Table 9).

### 3.10. Microbiological Analysis of Milk and Cheese

**Figure 3:**
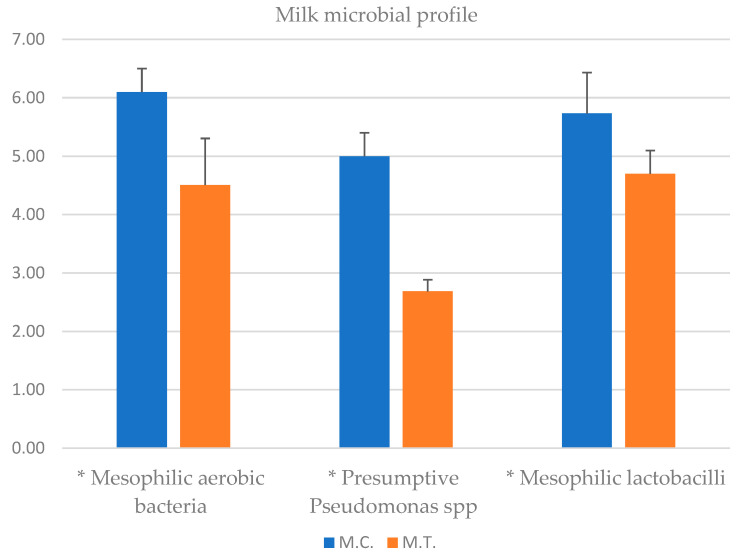
Milk microbial profile. Results are reported as mean ± standard deviation (n = 3) (log cfu/mL) of milk samples. M.C: milk control; M.T: milk treated; * *p*-value < 0.05.

**Figure 4:**
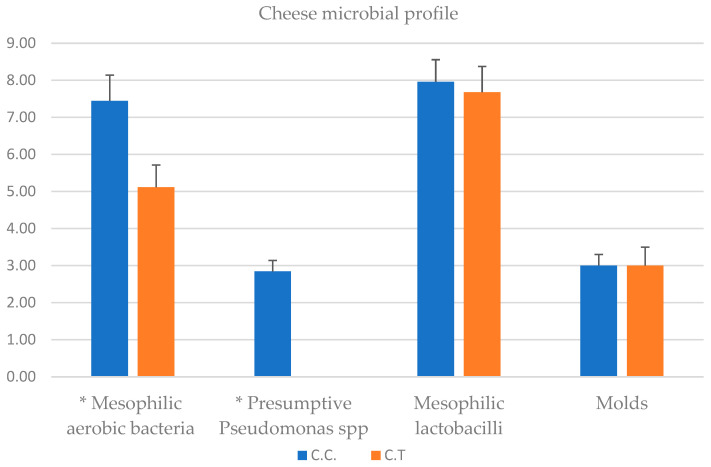
Cheese microbial profile. Results are reported as mean ± standard deviation (n = 3) (log cfu/g) of cheese samples. C.C: cheese control, C.T: cheese treated; * *p*-value < 0.05.

The results for microbiological analysis for milk samples are reported in Figure 3. Mesophilic aerobic bacteria (MAB) count in CTR milk was 6.10 ± 0.40 log_10_ CFU/mL and 4.51 ± 0.80 log_10_ CFU/mL for TRT milk (*p*-value < 0.05). Mesophilic lactobacilli count was 5.73 ± 0.4 log_10_ CFU/mL in CTR milk, 4.70 ± 0.2 log_10_ CFU/mL in TRT milk (*p*-value < 0.05). 5 ± 0.7 log_10_ CFU/mL of Presumptive *Pseudomonas* spp. were counted in CTR milk, and 2.69 ± 0.4 log_10_ CFU/mL in TRT milk (*p*-value < 0.05). *Listeria monocytogenes* and *Salmonella* were absent in 25 mL of milk. *Staphyloccus aureus* was not detected in milk (CTR and TRT) such as Total coliforms, Enterococci, Yeasts and Molds. Mesophilic aerobic bacteria count in CTR cheese was 7.44 ± 0.7 log_10_ CFU/g and 5.11 ± 0.6 log_10_ CFU/g for TRT cheese respectively (*p*-value < 0.05). Mesophilic lactobacilli count was 7.96 ± 0.6 log_10_ CFU/g in CTR cheese and 7.67 ± 0.7 log_10_ CFU/g in TRT cheese. 2.84 ± 0.3 log_10_ CFU/g of Presumptive *Pseudomonas* spp. were counted in CTR cheese, but they were not detected in TRT cheese (*p*-value < 0.05). Molds counts were 3 log_10_ CFU/mL for CTR (±0.3) and TRT (±0.5) cheeses (*p*-value > 0.05). *Listeria monocytogenes* and *Salmonella* were absent in 25 g of cheese. *Staphyloccus aureus*, Total coliforms, Enterococci and Yeasts were not detected in both cheeses (CTR and TRT) (Figure 4).

### 3.11. Quantitative Descriptive Analysis (QDA) of Cheese at 30 Days

**Figure 5:**
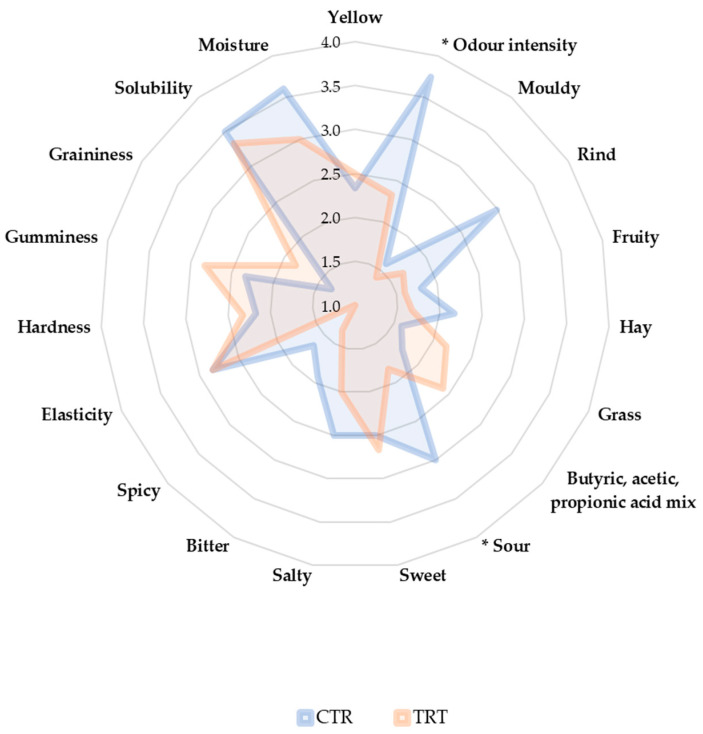
Radar of quantitative descriptive analysis of cheese at 30 days. CTR: control cheese; TRT: experimental cheese; 30 days of ripening; * *p*-value < 0.05 for odour intensity and sour attributes; (n = 3).

Sensory analysis on cheeses by QDA provided statistical differences between CTR and TRT tested after 30 days of ripening. The panellists detected lower odour intensity and sour in TRT cheese (*p*-value < 0.05). No differences were highlighted for other attributes of colour, odour, taste and texture (*p*-value > 0.05) (Figure 5).

### 3.12. Pearson’s Correlation of Cheese Variables

**Figure 6:**
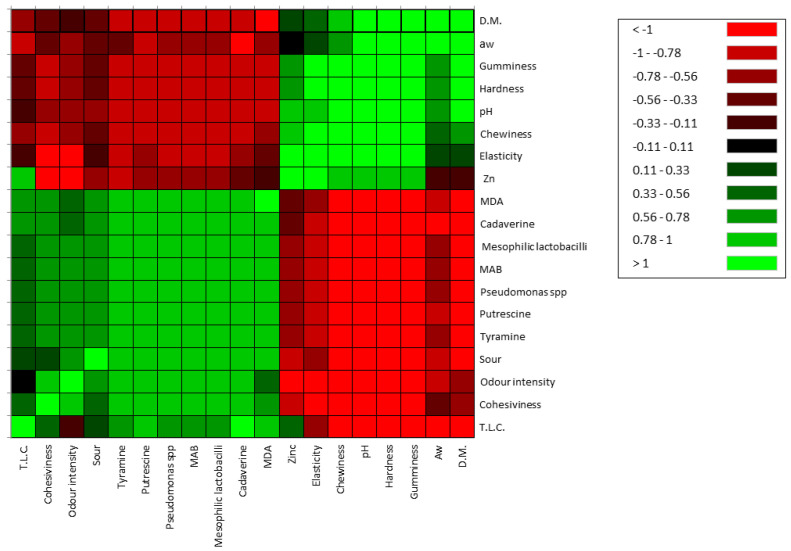
Correlation Heatmap of selected variables of cheese at 30 days. T.L.C: Total lipid content; MAB: mesophilic aerobic bacteria; D.M: dry matter; green colour represents positive correlation, (range: 0–1); red colour represents inverse correlation (range: −1, 0).

It was possible to correlate some studied variables by Pearson’s correlation. Zinc was directly correlated with Elasticity (0.777), pH (0.467), total lipid content (0.417), Hardness (0.474), Gumminess (0.469), and Chewiness (0.467). Some inverse correlations were highlighted for a_w_ (−0.083)_,_ dry matter (−0.097), Cohesivity (−0.583), cadaverine (−0.172), putrescine (−0.304) and tyramine (−0.438), MDA (−0.071), sour (−0.399), odour intensity (−0.807), MAB (−0.371), Mesophilic lactobacilli (−0.371) and presumptive *Pseudomonas* spp. (−0.371). Hardness was correlated with pH (0.938), zinc content (0.474), dry matter (0.723); Cohesivity with cadaverine (0.725), putrescine (0.781), tyramine (0.819) and MDA (0.661); Gumminess with pH (0.948), dry matter (0.743), Hardness (0.999), Elasticity (0.741) and Chewiness (0.880); Elasticity with pH (0.619), Hardness (0.761), Gumminess (0.741), and Chewiness (0.846). Biogenic amines are correlated primarily with MAB, mesophilic lactobacilli, presumptive *Pseudomonas* spp., total lipids content and cohesivity (range 0.5–1) (Figure 6).

## 4. Discussions

Zn supplied to lactating cows in different concentration can have varying effects on milk chemical. Some studies found that Zn supplementation did not significantly affect its chemical composition [5,6,7]. In ruminants, zinc absorption corresponds to about 15% of dietary Zn and occurs mainly in enterocytes (NRC, 2001). In the rumen, free ions of Zn^2+^ can form insoluble complexes, or constitute a source of nutrition for microorganisms by reducing the amount of Zn available in the intestine [25]. The solubility of trace minerals in the rumen can affect its absorption in the small intestine. If they becomes insoluble in the ruminal environment due to its binding nature, its availability for absorption may be reduced, in contrast to forms of trace minerals that either remain soluble or never become soluble in the rumen [26].

Zn metabolism is known to be regulated by changes in absorption and fecal endogenous excretion, as documented by [27]. When the intake of Zn is insufficient compared to the requirements, the efficiency of absorption and retention of zinc increases. On the other hand, when Zn intake exceeds the requirements, the absorption of Zn decreases and there is an increase in fecal endogenous excretion, all in an effort to maintain zinc concentrations in the body within a narrow range [28].

Fatty acids composition (FA) was affected by Zn in an investigation of [15], in which zinc was integrated for a content of 100 mg/kg.

The cows received about 22 kg head/day of dry matter of TMR but with different composition from that we utilized. A reason for this difference could be attributed to different diet composition. In our study integrating 120 mg/kg of ZnO no statistically significant differences were found in fatty acids profile. The same results were obtained for Zn content in milk and cheese. Also in the study of [15] no increase was detected in milk and cheese matrices. Contrary to a study of [5], where the authors observed a significant increase of Zn concentration following to organic Zn supplementation (Zn AA complex chelated by Zn-lysine and Zn-glutamic acid) with zinc content corresponding to 140 mg/kg. In our case we integrated zinc in inorganic form. This could be the difference but there could be some possible biochemical explanations. Zn transporter 4 (ZnT4) is abundant in cells surrounding the alveolar ducts in the mammary gland [29]. Huang et al., (1997) [30] following a study of a lethal milk mutation, a nonsense mutation at arginine codon 297 in the ZnT4 gene producing an incomplete translated ZnT4 protein, suggested that ZnT4 plays a critical role in determining the zinc content secreted in milk. In their study [30,31] showed ZnT4 refractory expression to different levels of zinc intake. It is also important to highlight the % of unabsorbed dietary Zn that could affect secreted zinc content. The absorption of Zn also relies on presence of other nutrients.

Analysis of fatty acids profile of cheese did not show some significant differences, as expected from milk FA composition. In contrast [15] observed that dietary ZnO integration (100 mg/kg) decrease concentration of satured fatty acids and increase oleic, vaccenic and rumenic acids.

Regarding lipid oxidation, Zn supplementation improved oxidative stability of cheese (Zn). CTR and TRT fresh cheese differed significantly for TBARS content. These differences increased over the ripening period. Considering the difference in fresh samples it is evident zinc effect in milk synthesis and its antioxidant effect in mammary glands. Zn has been reported to inhibit free radical lipid peroxidation in biological systems through a mechanism which involves the prevention of OH• and O_2_^−^ production. In particular, Zn has not been proven to directly interact with reactive oxygen species or with carbon-centered free radicals but it has been demonstrated to exert an antioxidant effect by competing with prooxidant metals (i.e., Cu and Fe) for binding sites, thus decreasing their ability to transfer electrons in a particular environment [32]. Zago et al., (2000) [33] demonstrated that in vitro zinc can protect membranes from Fe^2+^-initiated lipid oxidation, possibly by occupying iron binding sites. Zinc can also protect membranes from the deleterious effect of metals such as aluminium that induce changes in the physical properties of lipid bilayers. Zinc could have an important role as an antioxidant in biological systems and may interact with other components of the oxidant defense system.

Zinc supply to cows can be crucial for milk lipolysis. Hermansen et al., (1995) [34] found that adding Zn did not immediately affect free fatty acids in milk, but reduced their formation during cold storage. In their investigation Zn supplementation increased zinc concentration in cream, potentially impacting milk fat globule membrane suggesting when zinc intake is low, milk lipolysis risk increases. Zinc is also known to acting as an antioxidant together with metallothioneins (MTs), a protein family abundant in cysteine groups that plays a significant role in various physiological and pathological processes, particularly oxidative stress. One crucial function of MTs is their ability to tightly bind zinc and act as a pool for intracellular zinc preventing deficiency and maintaining optimal levels in animal tissues. The expression of MTs is stimulated by an increase in zinc levels, ensuring the maintenance of zinc homeostasis in biological systems. The fact that MTs not only have potent radical scavenging effects but also mediates the effects of zinc underscores their critical involvement in oxidative stress [1]. Similar results for lipid oxidation in cheese were found by [35,36].

Dry matter content of cheese was affected by Zn integration. During ripening period the CTR cheese lose 2% of humidity, less than TRT (4%). Water in cheese exists in two forms: bound and free. Bound water is responsible for hydrating hydrophilic molecules and dissolving solutes, but it is not accessible for biological functions [37]. a_w_ at a specific temperature is determined by dividing the equilibrium partial vapor pressure of water in the system by the equilibrium partial vapor pressure of pure liquid water at that same temperature [38] and it represents the free water. a_w_ plays a crucial role in food science, impacting safety and shelf life by influencing microbial growth and moisture migration. It characterizes the water’s energy level of food representing one of the most important intrinsic properties to determine the water capacity to be involved in chemical and biochemical reaction and for predicting the survival of microorganisms in a food product. The lower limit of available water for microbial growth is determined by a_w_, not water content. Most food spoilage bacteria will grow at a minimum water activity of approximately 0.90. The values reported in our studies allowed the microbial growth. The initial a_w_ (0.99) decreases of 0.01 in both cheese without significant variations.

Also pH was significantly affected by treatment. Variations in pH values during maturation can be attributed to different chemical compound produced by microbial metabolism and proteolysis by microbial or endogen enzymes. The development of microorganisms in cheese matrix is associated with the degradation of proteins, carbohydrates and lipids. At 30 days, it was observed that in the CTR cheese, there was a higher microbial count of MAB and mesophilic lactobacilli. The pH after one day of production (T0) experienced a slight but significant increase of 0.2 pH units. Indeed, in this study pH is correlated negatively with MAB, mesophilic lactobacilli and with biogenic ammine production. Considering no significant differences in milk pH reported, it is likely that microbial development and proteolysis led to this parameter increase. It can be observed that in the CTR cheese, there is a lower percentage of κ-casein and a higher percentage of bands n.4 and n.9. These bands may correspond to peptides of lower molecular weight. Experimental cheese (T0 and T30) showed better resistance to proteolysis. Zinc ions can form complexes with αS1-, β- and κ-caseins thanks to phosphate groups, carboxylic groups of Glu and Asp, and aromatic amino acids such as Tyr, Trp and Phe [39] and can be retentate in the curd during milk coagulation and it could inhibit some endogen proteases such as metalloproteinases (MMPs), zinc-dependent enzymes. Nosrati et al., (2019) [40] proposed that Zn may suppress MMPs expression and activity but the inhibition seems to depend on physiological conditions of the animals used, dose of Zn used, and duration of treatment. Also their tissue inhibitors are zinc dependent (TIMPs) and zinc can inhibit the activity of MMPs [41]. Another reason for this difference could be due to reduced residual activity of coagulant enzymes during the first days of maturation. Zn could have inhibited chymosin and plasmin in TRT cheese.

Analysis on milk proteic profile showed no differences, differently to cheese. Caseins are susceptible to proteolitic events, in particular α-caseins at the end of ripening were subjected to greater proteolysis in Ctr cheese for the effect of interaction of treatment and time. Other two proteic fragments increased their %. We could not identify them but the majority of peptides within the molecular weight range of 10–20 kDa were produced through the hydrolysis of αS-CN and β-CN by rennet and plasmin [42]. TRT cheese showed lower extent of proteolysis during the ripening period. Degradation of αs1- and β-casein during cheese maturation can be addressed to residual activity of both chymosin and plasmin [43]. Proteases can also have microbial origin. In our samples no starter was utilized. Cheese fermentation and maturation were carried out by natural milk microbiota, such as lactic acid bacteria (LAB) that could be affected by zinc presence [44]. Within bacteria that can release during cold storage of milk and cheese proteases there are *Pseudomonas* spp. [45]. Their higher presence in CTR cheese could have contributed to higher proteolitic degradation of caseins.

The texture of cheese is influenced by a combination of factors, including milk and cheese composition, manufacturing techniques, and ripening conditions which interact in a complex manner [46]. pH and the ratio of casein to moisture represent one of the most important factors. Zn affected textural properties of cheese (hardness, gumminess, chewiness, elasticity) with higher values in fresh and ripened TRT cheese. Cheese consists of a hydrated protein matrix with dispersed fat globules, together contribute to the structural properties. A strong network, more resistant to proteolytic reactions, allows to maintain a better hardness. Cheese softening occurs due to proteolysis caused by residual chymosin and native milk enzymes, along with proteases from starter cultures and other microorganisms [47]. The hydrolysis of a small portion of αs1-casein resulting in an overall reduction in the strength of the casein structure. During the ripening, a complex balance occurs between the proteolysis and hydration of the casein strands that tend to reduce the hardness of the cheese. However, the loss of moisture during this process leads to an increase in protein concentration, exerting an opposite effect on hardness [43]. Free water contributes to determining the a_w_ and the hardness of cheese increases with decreasing humidity. The greatest decrease of moisture was observed in TRT cheese. It also showed lower proteolysis than CTR, in which a significant decrease in hardness was observed, reflecting a faster protein degradation. A better balance between the reduction of water content and proteolitic events led to a natural increase of hardness. The significant differences at 1 day remained until 30 days increasing their values in TRT cheese. The increase of textural parameters, except for the cohesivity, reflects the natural aging process of cheese maturation. In this work we observed a high number of MAB and mesophilic lactobacilli in the control milk group, that is not necessarily correlated with mastitis in the cow control group; in this regard, studies of [48], using metagenomic analysis have evidenced that in addition to mastitis pathogens, which usually have a variety of virulence factors that enable them to resist the defence mechanisms of the udder, a diverse range of opportunistic and commensal bacteria can also inhabit the intramammary ecosystem. The microbiological analysis of milk showed a significant reduction in the bacterial count of the principal microbial groups (MAB, mesophilic lactobacilli and Presumptive *Pseudomonas* spp.) present in milk samples deriving from cow feeding with zinc oxide supplementation. These bacterial groups are commonly present in the milking environment and can penetrate the udder through the milking process. The reduction observed can be seen as advantageous because of their capacity to release high-temperature stable lipase and proteases can degrade proteins and fats [45]. In fact, they can cause milk spoilage after prolonged periods of cold storage. The reduction of the bacteria observed could attributed to the fact that: (i) Zinc may improve the cell immune functionality, and favouring the presence of microorganisms that produce a wide range of bacteriocins capable to inhibit the growth of some bacterial groups [49]; (ii) an increase of milk compounds with antimicrobial activity; in this context, Ref. [7] observed an increase of Ceruloplasmin (CP), by integrating zinc in cow diet. CP is a blue plasma alpha-2-glycoprotein that binds 90 to 95% of plasma copper [50] that belongs to the family of multicopper oxidase. CP is believed to play an important role in iron metabolism and homeostasis as it allows the incorporation of Fe3+ into apo-transferrin and is known to form complex with lactoferritin [51] which has antimicrobial properties against Gram-positive and Gram-negative bacteria, yeast and parasites [52]. Very interesting resulted the absence of Presumptive *Pseudomonas* group in TRT cheese after 30 days of storage. It is important to underline that *Pseudomonas* spp. undergo evolution as either planktonic or biofilm form within intricate microbial ecosystems found in cheese matrices, which are exposed to diverse physicochemical environments. It is well known that lactic acid bacteria (LAB) are largely predominant bacteria in cheese and numerous strains have been associated with food systems produce bacteriocins, that a wide inhibitory spectra against Gram negative bacteria included *Pseudomonas* spp. [53]. Thus, as we hypothesise before, it could be possible that the reduction this group in cheese could be also due to the presence of particular strains of LAB bacteriocin producers. By Pearson’s correlation some important relations between investigated variables are found. Inverse and direct relationship were obtained. Texture parameters, pH, A_w_, dry matter, biogenic amines production, oxidative state were highly affected by zinc treatment. The most correlated parameters that have been influenced by the zinc content have been those relating to pH, texture, microbial growth, the production of biogenic amines and the oxidative stability. MAB, mesophilic lactobacilli and *Pseudomonas* spp., negatively correlated with pH, hardness, dry matter, elasticity, gumminess, chewiness, zinc and a_w_, and positively with biogenic amines, total lipids content an oxidative stability. The pH affects hardness, gumminess, and elasticity. In the control cheese, the major and significant acidification had a clear effect on the decrease of the main parameters of the texture of the product showing an inverse relationship but confirming their interconnection.

Ripened cheeses have been linked to foodborne illnesses due to their elevated levels of biogenic amines (BAs), including tyramine, histamine, putrescine, and 2-phenylethylamine [54]. However, the content of these compounds can differ significantly between various cheese varieties and even within distinct sections of the same cheese. BAs are produced by indigenous microbiota with decarboxylase capabilities. The formation of BAs in food is associated with amino acid precursors, microorganisms that decarboxylate or aminate them [55], and favorable conditions for microorganism growth [56]. Other factors like storage conditions, temperature, and food production processes also influence their formation [57]. In fermented foods, lactic acid bacteria produce BAs to survive in acidic environments. Decarboxylation of amino acids happens faster in acidic conditions (pH 4.0–5.5) [58]. The activation of decarboxylative pathways is driven by various physiological factors. Notably, the decarboxylation of amino acids is linked to an electrogenic antiport system, which serves to counterbalance intracellular acidification [59]. As a result, the accumulation of BAs may serve as a cellular defense mechanism against acid stress. Some studies showed that the expression of numerous decarboxylase genes is upregulated in response to low pH, enhancing cell functionality in acidic environments [58]. Furthermore, the movement of a positive charge from inside the cell to the outside can create a proton motive force, resulting in the energization of the cell membrane and the provision of additional energy. Research has shown that the decarboxylase pathway can sustain primary metabolism during challenging environmental circumstances [60]. This feature may play a crucial role for microorganisms that do not have a respiratory chain, like the majority of LAB. It should be emphasized that the ability to generate BAs is typically unique to each strain, showing significant diversity in aminobiogenetic capacity among strains of the same species [54]. In our investigation a faster acidification occurred in CTR cheese. This phenomenon, together with the higher count of MAB and *Pseudomonas* spp. of milk and cheese could explain the higher BAs content. Conversely in the experimental cheese BAs content was under limit of detection (10 μg/g) both for fresh and ripened samples. In CTR cheese at 30 days a high content of cadaverine, putrescine and tyramine were determined. Putrescine can be formed with ornithine and agmatine as substrates, catalyzed by ornithine decarboxylase (EC: 4.1.1.17) and agmatinase (EC: 3.5.3.11), respectively. Tyramine is converted by tyrosine decarboxylase from tyrosine [54] and cadaverine can be formed by l-lysine. Putrescine and cadaverine can be produced from LAB [61] and *Pseudomonas* spp. [62,63] and tyramine from LAB [64]. Observing the different BAs content it is evident that zinc have affected microbial growth and could have affected mesophilic lactobacilli growth by selecting some strains. In a study of [44] the response of LAB to zinc fortification in milk was complex and strain-dependent with some strains more affected than others. For cheesemaking production we did not add starter culture, for that reason the mesophilic lactobacilli enumerated corresponding to non-starter lactic acid bacteria (NSLAB). These bacteria, usually found in raw milk cheese, are facultative heterofermentative lactobacilli [65] and they can produce a higher content of BAs in respect to starter LAB. In addition, a partial explanation for absence of BAs in TRT cheese could be given to the absence of *Pseudomonas* spp. It is possible to assume that in TRT cheese a decrease in the speed of all these biochemical reactions is mainly attributable to a lower count of MAB and *Pseudomonas* spp. either of raw milk and cheese. This could help us better explain the difference in the trend of aging at 30 days. According to the panellists, the sensory analysis highlighted lower odour intensity and sour in experimental cheese at 30 days. These results are in accordance with the less intensity of proteolitic events identified by electrophoretic analysis, with the less acidification, less content of BAs and the better oxidative stability observed in TRT cheese. Regard the odour intensity the production of aromatic compounds of cheese is primarily attributed to the lipolytic and proteolytic pathways, as well as the biochemical processes involving lactose, lactate, and citrate [66]. The better stability of experimental cheese could give an extended shelf life of this product, highlighting the importance of adequate levels of minerals in cow lactating diet.

## 5. Conclusions

ZnO supplementation reduced mesophilic aerobic bacteria and Presumptive *Pseudomonas* spp. growth, proteolysis, biogenic amines content, lipid oxidation, odour intensity and sour and increased hardness, gumminess, chewiness, elasticity of caciotta cheese. Biochemical and microbial reactions that were took place during ripening period represent an important starting point to evaluate the possibility to analyse the metagenomic profile of cheese investigated. Further investigation is required to gain a comprehensive understanding of the intricate relationships between zinc supplementation in animals, its influence on animal product composition, and ultimately, its implications on human health. In conclusion, Zn integration in cow lactating diet at 120 mg/kg could represent a promising strategy for improving the quality and safety of caciotta cheese and extending its shelf life.

## Figures and Tables

**Table 1:** Total mixed ration ingredients and chemical composition administered to CRT and TRT group.

	CTR	TRT
Total mixed ration ingredients		
Hay (%)	68.2	68.2
Concentrate (%)	13.6	13.6
Bread (%)	9.1	9.1
Barley flour (%)	4.1	4.1
Wheat flour (%)	4.1	4.1
Supplement 1 (%)	0.5	0.5
Supplement 2 (%)	0.5	0.5
Total mixed ration chemical composition (%)		
Dry Matter	56.2	56.2
Crude protein	14.4	14.4
Crude fat	3.02	3.02
Crude Ash	5.72	5.72
Neutral detergent fiber	31.53	31.53
Acid detergent fiber	21.66	21.66

Data are reported as mean (n = 3); CTR: control group, TRT: experimental group; Total mixed ration chemical composition (%) is expressed on dry matter basis. Supplement 1 ingredients: Potassium carbonate, magnesium carbonate, sodium chloride, polyphenols from hydrolyzed lignocellulose, flowers and leaves of *Filipendula U. Boswella Serrata*, common wheat. Supplement 2 ingredients: Calcium carbonate from crushed lime rocks (Ca 38%), sodium bicarbonate, magnesium oxide, sodium chloride, soluble wheat distillates, common wheat.

**Table 2:** Concentrate chemical composition and fatty acids profile.

	CTR C	TRT C
Concentrate chemical composition (%)		
Dry Matter	88.7	88.7
Crude protein	18.0	18.0
Crude fat	4.50	4.50
Crude fiber	7	7
Crude Ash	8	8
Sodium	0.35	0.35
Zinc (mg/kg)	38	120
Fatty acids (%)		
C16:0	21.09	21.09
C18:0	3.54	3.54
C20:0	0.22	0.22
C 22:0	0.10	0.10
C18:1, c9	23.54	23.54
C18:2, c9, c12	45.19	45.19
C18:3, c9, c12, c15	3.37	3.37
SFA	24.95	24.95
MUFA	23.54	23.54
PUFA	48.56	48.56
Others	2.95	2.95

Data are reported as mean (n = 3); CTR C: concentrate of control group; TRT C: concentrate of experimental group; Fatty acids are reported as means of relative percentage of total fatty acids; Concentrate chemical composition: The values are expressed as percentage on 100 g/dried weight and zinc content is expressed a mg/kg with a moisture content of 12%; Ingredients: Maize flour, meal based on dehulled soya flour (beans), barley flour, sunflower, husked extraction flour, wheat screening, sugar cane molasses, calcium carbonate, soya oil, sodium bicarbonate, sodium chloride.

**Table 3:** Milk chemical composition.

	CTR M	TRT M	*p*-Value
Caseins (%)	2.76 ± 0.01	2.66 ± 0.01	0.06
Lactose (%)	4.79 ± 0.04	4.86 ± 0.05	0.29
Lipids (%)	4.55 ± 0.03	3.96 ± 0.03	0.07
Proteins (%)	3.68 ± 0.01	3.56 ± 0.01	0.08
Urea (mg/L)	27.49 ± 1.27	28.13 ± 0.01	0.68
pH	6.69 ± 0.01	6.73 ± 0.01	0.21
Zinc (mg/kg)	3.7 ± 0.1	3.8 ± 0.2	0.83
C4:0	2.64 ± 0.14	3.04 ± 0.92	0.60
C6:0	2.11 ± 0.08	2.39 ± 0.63	0.59
C8:0	1.46 ± 0.05	1.59 ± 0.37	0.67
C10:0	3.55 ± 0.10	3.81 ± 0.59	0.60
C12:0	4.32 ± 0.09	4.60 ± 0.56	0.56
C14:0	14.37 ± 0.56	14.85 ± 0.14	0.65
C15:0	0.48 ± 0.00	0.48 ± 0.02	0.83
C16:0	36.83 ± 0.55	35.97 ± 0.46	0.23
C18:0	7,82 ± 0.46	7.68 ± 0.01	0.88
C20:0	0.19 ± 0.04	0.17 ± 0.05	0.71
C14:1	1.75 ± 0.06	1.65 ± 0.05	0.21
C16:1	2.95 ± 0.06	2.23 ± 0.08	0.13
C18:1 trans11	0.56 ± 0.08	0.74 ± 0.11	0.19
C18:1 cis 9	19.04 ± 0.03	18.22 ± 0.22	0.65
C18:2	1.52 ± 0.00	1.59 ± 0.22	0.70
C18:3	0.28 ± 0.21	0.15 ± 0.03	0.48
SFA	73.78 ± 0.16	74.59 ± 0.70	0.71
MUFA	23.41 ± 0.05	22.84 ± 0.36	0.77
PUFA	1.80 ± 0.21	1.74 ± 0.24	0.81
CLA	1.02 ± 0.10	0.82 ± 0.10	0.19
SCFA	4.75 ± 0.21	5.43 ± 0.54	0.60
MCFA	32.96 ± 0.62	33.60 ± 0.93	0.79
LCFA	69.31 ± 0.17	67.58 ± 0.27	0.62
ΔC14	0.11 ± 0.00	0.10 ± 0.00	0.11
ΔC16:1	0.05 ± 0.00	0.06 ± 0.00	0.09
ΔC18:1	0.71 ± 0.01	0.70 ± 0.00	0.59
ΔCLA	0.64 ± 0.06	0.53 ± 0.01	0.09
AI	3.91 ± 0.15	4.10 ± 0.62	0.72
TI	6.24 ± 0.06	6.38 ± 0.23	0.49

Data are reported as mean ± standard deviation (n = 3); Fatty acids are reported as means of relative percentage of total fatty acids; CTR M: control milk; TRT M: experimental milk; ns = not significant (*p* > 0.05); SFA = Saturated Fatty Acids; MUFA = Monounsaturated Fatty Acids; PUFA = Polyunsaturated Fatty Acids; CLA = Conjugated Linoleic Acids; Δ = Desaturation Index. ΔC14 = C14:1/(C14:1 + C14:0); ΔC16:1 = C16:1/(C16:1/C16); ΔC18:1 = C18:1/(C18:1 + C18:0); ΔCLA = CLA/(CLA + C18:1, t11); ΔCLA = (CLA/(C18:1, t11 + CLA)] × 100; AI = (C12:0 + 4 × C14:0 + C16:0)/(MUFA + PUFA(n3, n6); TI = (C14:0 + C16:0 + C18:0)/(0.5 × MUFA) + (0.5 × PUFAn6) + (3 × PUFAn3) + (n3/n6).

**Table 4:** Cheese fatty acids profile.

	CTR	TRT	CTR	TRT	*p*-Value			RMSE
	T0		T30		Z	T	Z × T	
C4:0	3.57	3.09	3.15	3.42	0.90	0.96	0.67	1.53
C6:0	2.86	2.50	2.40	2.67	0.94	0.81	0.59	1.06
C8:0	1.94	1.73	1.57	1.84	0.93	0.71	0.50	0.63
C10:0	4.55	4.16	3.71	4.29	0.89	0.60	0.49	1.24
C12:0	4.95	4.70	4.19	4.71	0.78	0.46	0.45	0.90
C14:0	14.52	14.36	13.35	14.43	0.54	0.46	0.41	1.33
C15:0	1.73	1.61	1.64	1.60	0.08	0.14	0.24	0.05
C16:0	33.58	33.98	34.32	33.69	0.90	0.81	0.59	1.70
C18:0	7.52	7.89	8.51	7.68	0.78	0.64	0.48	1.48
C20:0	0.19	0.20	0.25	0.19	0.47	0.52	0.32	0.06
C14:1	2.22	2.31	1.80	2.33	0.07	0.22	0.18	0.28
C16:1	2.21	2.26	2.31	2.25	0.96	0.52	0.47	0.13
C18:1 trans 11	0.57	0.71	0.66	0.66	0.26	0.75	0.25	0.11
C18:1 cis 9	16.93	17.70	19.29	17.40	0.73	0.53	0.42	2.88
C18:2	1.38	1.53	1.50	1.52	0.46	0.63	0.59	0.21
C18:3	0.46	0.39	0.43	0.42	0.60	0.99	0.65	0.12
SFA	75.41	74.23	73.08	74.50	0.95	0.59	0.49	0.82
MUFA	21.93	22.98	24.05	22.65	0.92	0.59	0.46	0.80
PUFA	1.84	1.92	1.93	1.95	0.76	0.74	0.85	0.95
CLA	0.83	0.87	0.94	0.90	0.96	0.29	0.56	0.63
SCFA	6.43	5.59	5.55	6.09	0.92	0.90	0.64	0.96
MCFA	29.91	28.88	26.24	29.20	0.69	0.49	0.41	0.70
LCFA	63.65	65.53	68.20	64.71	0.83	0.62	0.48	0.83
ΔC14	0.13	0.14	0.12	0.14	0.08	0.36	0.31	0.22
ΔC16:1	0.06	0.06	0.06	0.06	0.82	0.12	0.32	0.26
ΔC18:1	0.69	0.69	0.69	0.69	0.96	0.81	0.80	0.99
ΔCLA	0.59	0.55	0.59	0.58	0.06	0.22	0.06	0.02
AI	4.18	3.92	3.54	4.01	0.82	0.54	0.43	0.76
TI	6.19	6.15	5.92	6.18	0.41	0.38	0.29	0.48

All data are reported as means of relative percentage of total fatty acids (n = 3); CTR: control cheese; TRT: experimental cheese; T0: time 0; T30: 30 days of ripening; (Z) zinc treatment, (T) time and (Z × T) time and zinc interaction. RMSE: root mean square error. SFA = Saturated Fatty Acids; MUFA = Monounsaturated Fatty Acids; PUFA = Polyunsaturated Fatty Acids; CLA = Conjugated Linoleic Acids; Δ = Desaturation Index. ΔC14 = C14:1/(C14:1 + C14:0); ΔC16:1 = C16:1/(C16:1/C16); ΔC18:1 = C18:1/(C18:1 + C18:0); ΔCLA = CLA/(CLA + C18:1, t11); ΔCLA = (CLA/(C18:1, t11 + CLA)] × 100; AI = (C12:0 + 4 × C14:0 + C16:0)/(MUFA + PUFA(n3, n6); TI = (C14:0 + C16:0 + C18:0)/(0.5 × MUFA) + (0.5 × PUFAn6) + (3 × PUFAn3) + (n3/n6).

**Table 5:** Dry matter, pH, zinc and total lipid content of cheese.

	CTR	TRT	CTR	TRT	*p*-Value			RMSE
	T0		T30		Z	T	Z × T	
Dry matter	55.86 ^c^	55.49 ^b^	57.25 ^b^	59.19 ^a^	0.00 **	0.00 **	0.48	1.03
pH	4.85 ^a^	4.60 ^b^	4.24 ^c^	4.60 ^b^	0.00 *	0.00 **	0.00 **	0.03
a_w_	0.99 ^a^	0.99 ^a^	0.98 ^a^	0.98 ^a^	0.7	0.65	0.49	0.01
Zinc (mg/kg)	23 ± 2	24.3 ± 1.1	-	-	0.46	-	-	-
Total lipids content	41.01 ^b^	39.96 ^b^	48.63 ^a^	45.58 ^ab^	0.30	0.00 *	0.58	2.85

All values are reported as mean of 3 repetitions with standard deviation for zinc and root mean square error (RMSE) for dry matter, pH and total lipid content. Total lipid content is reported on base of dry matter. CTR: control cheese; TRT: experimental cheese; T0: time 0; T30: 30 days of ripening; (Z) zinc treatment, (T) time and (Z × T) time and zinc interaction. * *p*-value < 0.05; ** *p*-value < 0.001; different letters in the same row indicate statistically significant differences by interaction of Z × T.

**Table 6:** Densitometric analysis of SDS-PAGE of milk.

	CTR	TRT	*p*
band (%)			
α-casein	31.20 ± 1.09	30.28 ± 1.45	0.35
β-casein	19.45 ± 2.45	20.78 ± 2.97	0.51
κ-casein	8.57 ± 2.20	8.01 ± 2.68	0.76
Band n.4	2.21± 0.74	1.81 ± 0.75	0.48
Band n.5	4.04 ± 0.45	3.96 ± 1.37	0.91
β-lactoglobulin	22.94 ± 0.96	22.38 ± 0.79	0.40
α-lactalbumin	9.16 ± 0.47	9.97 ± 1.37	0.31
Band n.8	2.43 ± 0.29	2.81 ± 0.40	0.17

Data are reported as mean of relative percentage (n = 3) ± standard deviation; CTR: control milk; TRT M: treated milk.

**Table 7:** Densitometric analysis of SDS-PAGE of cheese.

	CTR	TRT	CTR	TRT	*p*-Value			RMSE
	T0		T30		Z	T	Z × T	
band (%)								
α-casein	36.81^a^	36.78 ^a^	21.61 ^c^	24.89 ^b^	0.00 *	0.00 **	0.00 *	0.90
β-casein	28.93 ^a^	28.69 ^ab^	26.11 ^b^	27.80 ^ab^	0.29	0.01 *	0.17	1.31
κ-casein	4.24 ^a^	5.40 ^a^	3.70 ^a^	4.07 ^a^	0.18	0.11	0.47	1.07
Band n.4	7.00 ^a^	6.67 ^b^	14.00 ^a^	11.78 ^a^	0.27	0.00 **	0.41	2.23
Band n.5	3.79 ^a^	4.43 ^a^	3.77 ^a^	4.73 ^a^	0.10	0.76	0.72	0.90
β-lactoglobulin	3.58 ^a^	3.51 ^a^	3.44 ^a^	3.63 ^a^	0.85	0.98	0.69	0.63
Band n.7	5.40 ^b^	5.78 ^b^	11.39 ^a^	10.53 ^a^	0.35	0.00 **	0.02 *	0.48
α-lactalbumin	4.91 ^a^	4.79 ^b^	10.36 ^a^	8.39 ^ab^	0.25	0.00 **	0.30	1.72
Band n.9	5.33 ^a^	3.92 ^a^	5.60 ^a^	4.17 ^a^	0.15	0.79	0.99	1.87

Data are reported as mean of relative percentage (n = 3); RMSE: root mean square error; CTR: control cheese; TRT: experimental cheese; T0: day 1; T30: 30 days of ripening; (Z) zinc treatment, (T) time and (Z × T) time and zinc interaction. * *p*-value < 0.05; ** *p*-value < 0.001; different letters in the same row indicate statistically significant differences by interaction of Z × T.

**Table 8:** Texture analysis of cheese.

	CTR	TRT	CTR	TRT	*p*-Value			RMSE
	T0		T30		Z	T	Z × T	
Hardness (N)	2.45 ^bc^	2.91 ^ab^	2.32 ^c^	3.49 ^a^	0.00 **	0.35	0.01 *	0.76
Cohesivity (%)	73.45 ^b^	74.14 ^a^	71.67 ^b^	69.39 ^c^	0.03 *	0.00 **	0.00 **	2.31
Gumminess (N)	180.51 ^bc^	215.47 ^ab^	166.22 ^c^	241.97 ^a^	0.00 **	0.97	0.04 *	55.37
Elasticity (%)	84.83 ^b^	87.42 ^ab^	86.11 ^ab^	88.81 ^a^	0.00 **	0.09	0.94	3.89
Chewiness (mJ)	154.02 ^b^	188.55 ^a^	135.51 ^b^	211.02 ^a^	0.00 **	0.84	0.04 *	50.34

Data are reported as mean (n = 3), RMSE = root mean square error; CTR: control cheese; TRT: experimental cheese; T0: time 0; T30: 30 days of ripening; (Z) zinc treatment, (T) time and (Z × T) time and zinc interaction. * *p*-value < 0.05, ** *p*-value < 0.001; different letters in the same row indicate statistically significant differences by interaction of Z × T. Cohesivity (%): Area 2 compression/Area 1 compression; Gumminess (N): Hardness (N) × Cohesivity; Elasticity (%): (Distance 2)/(Distance 1) × 100; Chewiness (mJ): Elasticity (mm) × Gumminess (N).

**Table 9:** BAs content of cheese.

	CTR	TRT	CTR	TRT
	T0		T30	
Putrescine	n.d	n.d.	50.46 ± 2.04	n.d.
Cadaverine	n.d	n.d.	229.52 ± 16.26	n.d.
Tyramine	n.d	n.d.	123.57 ± 4.56	n.d.
Tryptamine	n.d	n.d.	n.d	n.d.
2-Phenylethylamine	n.d	n.d.	n.d	n.d.
Histamine	n.d	n.d.	n.d	n.d.
Spermine	n.d	n.d.	n.d	n.d.
Spermidine	n.d	n.d.	n.d	n.d.

Data are reported as mean ± standard deviation based on dry matter (μg/g) (n = 3); CTR: control cheese; TRT: experimental cheese; T0: time 0; T30: 30 days of ripening; n.d.: not detectable; LOD: 10 μg/g.

## Data Availability

The data presented in this study are available on request from the corresponding author.
